# The moderated-mediation role of risk perception and intolerance of uncertainty in the association between residual symptoms and psychological distress: a cross-sectional study after COVID-19 policy lifted in China

**DOI:** 10.1186/s12888-024-05591-9

**Published:** 2024-02-16

**Authors:** Zhiyu Sun, Zhou Jin, Kejie Zhao, Xin Wen, Hui Lu, Nuonuo Hu, Qinxin Zhu, Yi Zhang, Minjie Ye, Yili Huang, Weihong Song, Deborah Baofeng Wang, Yili Wu

**Affiliations:** 1 Department of Psychiatry, The Second Affiliated Hospital and Yuying Children’s Hospital of Wenzhou Medical University, 325000 Wenzhou, Zhejiang China; 2 Zhejiang Provincial Clinical Research Center for Mental Disorders, Wenzhou Key Laboratory of Basic and Translational Research for Mental Disorders, School of Mental Health and The Affiliated Wenzhou Kangning Hospital, Key Laboratory of Alzheimer’s Disease of Zhejiang Province, Institute of Aging, Oujiang Laboratory (Zhejiang Lab for Regenerative Medicine, Vision and Brain Health), Wenzhou Medical University, 325000 Wenzhou, Zhejiang China; 3 Institute of Aging, Key Laboratory of Alzheimer’s Disease of Zhejiang Province, Zhejiang Provincial Clinical Research Center for Mental Disorders, Wenzhou Medical University, 325000 Wenzhou, Zhejiang China; 4 Zhejiang Provincial Clinical Research Center for Mental Disorders, Wenzhou Key Laboratory of Basic and Translational Research for Mental Disorders, School of Mental Health and The Affiliated Wenzhou Kangning Hospital, Wenzhou Medical University, 325000 Wenzhou, Zhejiang China; 5 Lyons Insights Consulting, 69534 Lyons, IL USA

**Keywords:** COVID-19, Residual symptoms, Psychological distress, Risk perception, Intolerance of uncertainty

## Abstract

**Background:**

A considerable number of individuals infected with COVID-19 experience residual symptoms after the acute phase. However, the correlation between residual symptoms and psychological distress and underlying mechanisms are scarcely studied. We aim to explore the association between residual symptoms of COVID-19 and psychological distress, specifically depression, anxiety, and fear of COVID-19, and examine the role of risk perception and intolerance of uncertainty in the association.

**Methods:**

A cross-sectional survey was conducted by online questionnaire-based approach in mid-January 2023. Self-reported demographic characteristics, COVID-19-related information, and residual symptoms were collected. Depression, anxiety, fear, risk perception and intolerance of uncertainty were evaluated using the Patient Health Questionnaire-9 (PHQ-9), Generalized Anxiety Disorder-7 (GAD-7), Fear of COVID-19 Scale (FCV-19S), COVID-19 Risk Perception Scale and Intolerance of Uncertainty Scale-12 (IUS-12), respectively. Linear regression analyses were conducted to explore the associations. A moderated mediation model was then constructed to examine the role of risk perception of COVID-19 and intolerance of uncertainty in the association between residual symptoms and psychological distress.

**Results:**

1735 participants effectively completed the survey. 34.9% of the patients experienced residual symptoms after acute phase of COVID-19. Psychological distress was markedly increased by COVID-19 infection, while residual symptoms had a significant impact on psychological distress (*Ps* < 0.001), including depression (β = 0.23), anxiety (β = 0.21), and fear of COVID-19 (β = 0.14). Risk perception served as a mediator between residual symptoms and all forms of psychological distress, while intolerance of uncertainty moderated the effect of risk perception on depression and anxiety.

**Conclusion:**

A considerable proportion of patients experience residual symptoms after acute phase of COVID-19, which have a significant impact on psychological distress. Risk perception and intolerance of uncertainty play a moderated-mediation role in the association between residual symptoms and depression/anxiety. It highly suggests that effective treatment for residual symptoms, maintaining appropriate risk perception and improving intolerance of uncertainty are critical strategies to alleviate COVID-19 infection-associated psychological distress.

**Supplementary Information:**

The online version contains supplementary material available at 10.1186/s12888-024-05591-9.

## Background

The novel coronavirus pneumonia has spread globally since 2020 [1], resulting in almost 701 million infections and approximately 6.9 million deaths by early 2024 [2]. The coronavirus disease 2019 (COVID-19) pandemic has significant detrimental effects on the global economy [3], physical health (severe acute syndrome [4] and sequelae [5]), as well as people’s daily lives [6, 7]. The waves of COVID-19 pandemic marked by the emergence of new variants and vaccination, e.g., the outbreak of the Omicron variant and Delta variant [8, 9].

COVID-19 pandemic is closely correlated with psychological distress, including depression, anxiety, and fear of COVID-19 etc., and its effect on mental health may persist over an extended period [10–15]. These adverse impacts on mental health may be even more evident among those with post-traumatic stress disorder caused by COVID-19 [16]. The transactional theory of stress and coping highlights the importance of the interplay between cognitive assessment and environment in the development of psychological distress during stressful events [17]. It indicates that psychological distress and associated factors may vary at different stages of the peri-infection period. Previous studies focus on the psychological distress either among general population or among patients with Long COVID-19 [5, 13–15, 18–20]. However, psychological distress and its aggravators throughout the COVID-19 infection process are less studied, particularly among patients experiencing acute remission of COVID-19.

The incidence of Long COVID-19, encompassing children, is estimated between 10 and 20%, predominantly manifesting in patients with mild acute symptoms [20]. Compared with Long COVID-19 which persists beyond one month following the initial acute syndrome of COVID-19 [18, 19, 21–23], more patients are accompanied with “residual symptoms” during acute remission of COVID-19. These symptoms may be more severe than Long COVID-19 and potentially increase the risk for developing Long COVID-19. Thus, the presence of residual symptoms may be associated with increased risk of psychological distress. However, the effect of residual symptoms on psychological distress and underlying mechanisms are scarcely studied [24].

Risk perception and intolerance of uncertainty (IU), two major factors involving in disease-associated psychological distress, may contribute to the effect of residual symptoms on psychological distress [25–27]. Risk perception of COVID-19 refers to an individual’s cognitive response, assessment, experience, and subjective feelings toward the risk associated with COVID-19 [28]. Residual symptoms following acute COVID-19 syndrome may indicate a prolonged negative impact on health [29], potentially leading to an increased perception of severity and persistence of COVID-19. The elevated levels of risk perception and appraisal may link to increased psychological distress [30]. Moreover, IU is a personal psychological trait that reflects a person’s inability to endure aversive responses, leading to negative reactions toward unpredictable situations or uncertain events, regardless of the probability of occurrence [31, 32]. For example, IU was a significant predictor of psychological distress during the COVID-19 pandemic [33]. It suggests that IU may potentially influence the connection between risk perception and psychological distress.

This study aimed to examine the psychological distress of individuals during the COVID-19 infection process, from high risk to contact the virus to infected within 1 month. Moreover, the effect of residual symptoms on psychological distress was examined, which fills the research gap between acute phase of COVID-19 and Long COVID. Furthermore, the moderated mediating effect of risk perception and IU on the relationship between residual symptoms and psychological distress was explored. Three hypotheses were proposed to achieve these objectives: (1) The level of psychological distress varies among individuals at different stages of COVID-19 infection; (2) Individuals with residual symptoms are more likely to experience more severe psychological distress; (3) The relationship between residual symptoms and psychological distress is mediated by risk perception and moderated by IU.

## Methods

### Study design and recruitment

This was a cross-sectional, descriptive and correlational study. Participants were categorized into different stages based on COVID-19 infection status, ranging from never being infected to fully recovery. The survey was conducted from January 12 to January 21, 2023. Most patients have recovered from acute phase of COVID-19 infection within this time window [34].

Convenience sampling was utilized in this study due to the unique nature of emergencies. Online recruitment was conducted in the form of Quick Response (QR) code through electronic questionnaires powered by “Questionnaire Star” (https://www.wjx.cn/). Participants were recruited using social media: WeChat and WeChat Moments. All participants were presented with study-related information and asked about consent preferences. The Ethics Committee of The Affiliated Kangning Hospital of Wenzhou Medical University approved this study (Approval Code: YJ-2023-16-01) following the Helsinki Declaration.

### Participants

A total of 1800 individuals completed the questionnaires. The questionnaires were individually checked by two investigators to eliminate those with extremely short filling times (less than 200s) or obvious random filling. Individuals who had been infected with COVID-19 for more than one month were excluded. 1735 completed questionnaires were included in the study. The exclusion rate was 3.61%.

### Measurements

#### Demographic factors and COVID-19-related information

Demographic factors were collected, including age, gender, religiosity, family financial situation, and physical health. COVID-19-related information was collected, including COVID-19 vaccination status, medicines preparation, financial losses during the pandemic and after lifting the COVID-19 policy, infection of relatives and friends, individual’s infection status and time of infection, recovery status, and any residual symptoms experienced after acute remission and nucleic acid turned negative.

#### Proposed stages of COVID-19 infection

The whole COVID-19 infection process was categorized into three stages based on the individual’s infection status (Supplementary Figure S1). The infection status was determined by asking, “Have you ever been infected with COVID-19?”, with three possible responses: (1) never, and do not exhibit any symptoms related to the virus such as fever, sore throat, cough, etc.; (2) never, but display suspicious symptoms related to the virus; and (3) have been infected with COVID-19 confirmed by a nucleic acid or antigen test. Participants who answered (1), (2), and (3) were categorized as stage 1, 2, and 3 groups, respectively. For participants who answered (3), an additional question “When were you first infected with COVID-19?” was asked. The response options were (1) within 1 week, (2) from 1 week to 1 month, and (3) over 1 month. Participants with answer (1) and (2) were clustered into ‘acute phase’ (stage 3a) and ‘chronic phase’ (stage 3c) of stage 3, respectively (Supplementary Figure S1). Participants with answer (3) were excluded from the current study.

#### Residual symptoms

COVID-19 related symptoms during the acute remission (within 1 month) were defined as residual symptoms, which differentiate from Long COVID (over 1 month). Residual symptoms should satisfy three criteria: (1) Individuals have been diagnosed with COVID-19 by nucleic acid or antigen detection; (2) Individuals have recovered from the acute syndrome and nucleic acid or antigen detection is negative; (3) COVID-19-related symptoms are still present within one month of infection.

To measure residual symptoms among participants who answered “have been recovered from COVID-19 acute syndrome and nucleus acid or antigen tests were negative”, the item ‘Do you still have symptoms (i.e., fever, cough, sore throat, stuffy nose, and fatigue)?’ was asked. Participants who answered ‘yes’ were categorized into the group with residual symptoms.

#### Risk perception of COVID-19

The COVID-19 Risk Perception Scale, developed by Cui Xiaoqian and colleagues, was used to assess the risk perception of COVID-19 [35]. The scale comprises nine items assessing three dimensions: susceptibility, severity, and controllability of COVID-19. Each item is rated on a scale of 1 to 5. Higher scores indicate higher levels of risk perception (total scores range from 9 to 45). The scale has good reliability and validity (α = 0.82), with an acceptable internal consistency (α = 0.90) in this study.

#### Depressive symptoms

The 9-item Patient Health Questionnaire (PHQ-9) was used to assess the levels of depressive symptoms in the last two weeks [36]. Each item was rated on a 4-point Likert scale ranging from 0 to 3. Higher scores indicate more severe depressive symptoms (total scores range from 0 to 27) [15, 16]. The reliability and validity of the Chinese version of PHQ-9 have been examined (α = 0.86) [37]. In this study, the internal consistency reliability was tested with α = 0.92.

#### Anxiety symptoms

The levels of anxiety symptoms in the last two weeks were assessed by using the 7-item Generalized Anxiety Disorder (GAD-7) scale developed by Spitzer and colleagues [38]. The response format for each item is like that of PHQ-9. The Chinese version of GAD-7 has been verified for reliability and validity (α = 0.90) [39]. Higher scores indicate greater severity of anxiety symptoms (total scores range from 0 to 21) [15, 16]. The internal consistency of the study was excellent (α = 0.95).

#### Fear of COVID-19

In this study, the Fear of COVID-19 Scale (FCV-19S) [40] developed by Ahorsu et al. was utilized to measure individuals’ specific fears of COVID-19. The FCV-19S comprises seven items, with each item rated on a 5-point Likert scale ranging from 1 to 5. The higher scores indicate greater fear of COVID-19 (total scores range from 7 to 35). The Chinese version of FCV-19S has shown good psychometric properties (α = 0.82) [41]. The internal consistency of the study was good (α = 0.94).

#### Intolerance of uncertainty

The current study utilized the short version of the Intolerance of Uncertainty Scale (IUS-12) to evaluate participants’ inclination towards uncertainty [42]. Each item is rated on a scale of 1 to 5. Higher scores indicate lower tolerance for uncertainty (total scores range from 12 to 60). The Chinese version of the IUS-12 has shown good reliability and validity (α = 0.88) [43], with reliable internal consistency (α = 0.94) in this study.

### Statistical analysis

The characteristics of psychological distress were described and psychological distress among participants at three different COVID-19 infection stages were evaluated. Analyses of covariance (ANCOVAs) and post hoc tests with the Bonferroni correction were used while the effects of age, gender, financial situation, and physical health were controlled as covariates. Linear regression analysis was applied to determine the univariate association between demographic factors as well as COVID-19-related information and psychological distress. Multiple regression analysis was conducted to analyze the contribution of residual symptoms in explaining psychological distress. Additionally, *t*-test was conducted to investigate the differences in risk perception of COVID-19 and psychological distress between subgroups of stage 3 as well as subgroups with and without residual symptoms after controlling covariates. SPSS 26.0 was applied for the analyses.

Structural Equation Modeling (SEM) was performed to test the moderated mediation by using Model 14 in the PROCESS macro version 3.3. The mediating role of risk perception in the association between residual symptoms and psychological distress was examined following partial correlation analysis. All continuous variables were standardized before testing the moderation. All regression coefficients were tested for significance using the bias-corrected percentile Bootstrap method [44]. The 5000 bootstrap samples were utilized to test hypothesized mediation and moderated effects.

## Results

### Demographic characteristics and COVID-19-related information

Demographic characteristics were shown in Table 1. The mean age of the included participants was 28.23 (SD = 13.84) years. 1181 participants (68.1%) were female and 524 (30.2%) had a religious affiliation. 208 participants (12.0%) rated their family’s financial situation as well-off. 129 participants (7.4%) reported having other health conditions such as chronic or serious disease or being in the perinatal period.

**Table 1:** Univariate associations between demographic characteristics, COVID-19-related information, and psychological distress (*n* = 1735)

Variables	Categories (n, %)	Depressive symptoms^a^		Anxiety symptoms^a^		Fear of COVID-19^a^	
		β	t	β	t	β	t
**Demographic characteristics**							
Age (mean ± SD)	28.23 ± 13.84	0.032	1.354	-0.009	-0.376	0.176***	7.444***
Gender	Female (*n* = 1181, 68.1%)	0.085***	3.549***	0.058*	2.435*	0.138***	5.788***
	Male (*n* = 554, 31.9%)	reference		reference		reference	
Religiosity	Secular (*n* = 1211, 69.8%)	0.003	0.116	0.002	0.102	-0.025	-1.023
	Religious/traditional (*n* = 524, 30.2%)	reference		reference		reference	
Financial situation	Relatively good (*n* = 208, 12.0%)	-0.113***	-4.740***	-0.083***	-3.465***	-0.147***	-6.174***
	Fair or poor (*n* = 1527, 88.0%)	reference		reference		reference	
Physical health	Other health conditions (*n* = 129, 7.4%)	0.133***	5.600***	0.133***	5.584***	0.084***	3.519***
	Health (*n* = 1606, 92.6%)	reference		reference		reference	
**COVID-19-related information**							
COVID-19 Vaccination	Booster dose (*n* = 734, 42.3%)	-0.020	-0.813	-0.021	-0.872	0.096***	4.028***
	0–2 dose (*n* = 1001, 57.7%)	reference		reference		reference	
Medicines preparation	Excessive or hoarding of medicines (*n* = 454, 26.2%)	-0.002	-0.085	0.004	0.157	0.020	0.833
	No or moderate preparation of medicines (*n* = 1281, 73.8%)	reference		reference		reference	
Financial losses during the COVID-19 pandemic	Loss (*n* = 1376, 79.3%)	0.152***	6.418***	0.131***	5.483***	0.143***	6.007***
	Without loss (*n* = 359, 20.7%)	reference		reference		reference	
Financial losses after lifting the COVID-19 policy	Loss (*n* = 1148, 66.2%)	0.143***	6.033***	0.132***	5.550***	0.192***	8.148***
	Without loss (*n* = 587, 33.8%)	reference		reference		reference	
Infections in relatives and friends	No (*n* = 208, 12.0%)	-0.063**	-2.627**	-0.051*	-2.115*	-0.025	-1.058
	Yes (*n* = 1527, 88.0%)	reference		reference		reference	
Residual symptoms (*n* = 802)	With residual symptoms (*n* = 280, 34.9%)	0.241***	7.023***	0.204***	5.896***	0.213***	6.180***
	No residual symptoms (*n* = 522, 65.1%)	reference		reference		reference	

a: Univariate linear regression analysis, SD: Standard deviation, β: Standardized coefficient, COVID-19: The coronavirus disease 2019, Other health conditions: Including chronic or serious disease or being in the perinatal period and so on, *: *P* < 0.05, **: *P* < 0.01, ***: *P* < 0.001

The COVID-19-related information is presented in Table 1. 734 participants (42.3%) received a booster vaccination and 454 (26.2%) had overprepared or hoarded medicines. Moreover, 1376 (79.3%) and 1148 (66.2%) participants reported experiencing financial losses during the COVID-19 pandemic and in the month following the lifting of the COVID-19 policy, respectively. Additionally, 1527 participants (88.0%) reported that their friends or relatives were infected with COVID-19. 822 (47.4%) participants had been infected with COVID-19 and were in stage 3. 596 (34.4%) participants were in stage 2, while 317 (18.3%) were in stage 1. Among 822 confirmed participants, 66 (8.0%) have been infected within a week (stage 3a), and 756 (92.0%) have been infected from 1 week to 1 month (stage 3c). Among the 802 individuals who have been relieved from the acute syndrome with negative nucleic acid, 280 (34.9%) experienced residual symptoms.

### Psychological distress

**Fig. 1:**
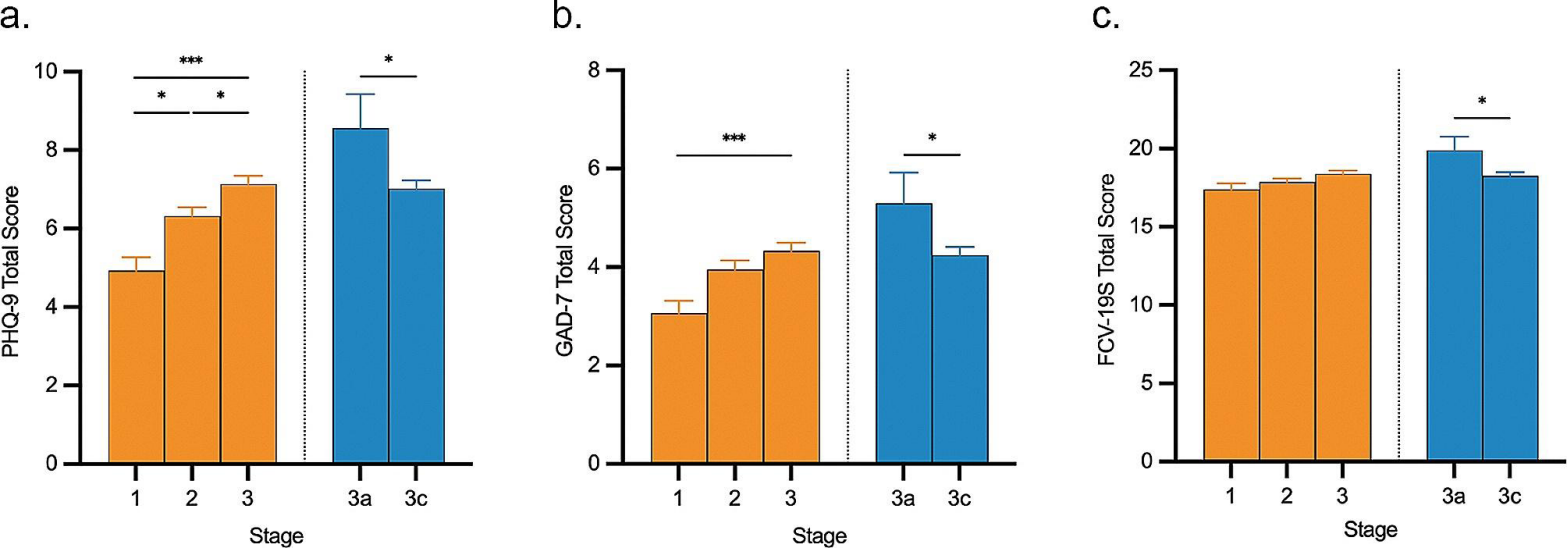
Psychological distress levels at different stages of COVID-19 infection. Depressive symptoms (**a**), anxiety symptoms (**b**) and fear of COVID-19 (**c**) were assessed by scales of PHQ-9, GAD-7 and FCV-19S, respectively. Stage 3a represents acute phase of stage 3, and stage 3c represents chronic phase of stage 3. Values represent mean ± SEM. *: *P* < 0.05, ***: *P* < 0.001 by one-way analyses of covariance (ANCOVAs) or Student’s t-test

The average scores for PHQ-9, GAD-7, and FCV-19S were 6.45 (SD = 5.88), 3.97 (SD = 4.68), and 18.02 (SD = 6.13), respectively, among all participants. As depicted in Fig. 1a, b and c, the levels of psychological distress tended to be higher in stage 2 relative to stage 1 and peaked in stage 3. Significant differences of depressive symptoms (F = 13.808, *P* < 0.001) and anxiety symptoms (F = 6.919, *P* = 0.001) were revealed among 3 stages, but no difference for fear of COVID-19 (F = 1.547, *P* = 0.213). The levels of depression and anxiety were significantly higher in stage 3 than those in stage 1 (*Ps* < 0.001). Moreover, the level of depression was significantly higher in stage 3 than that in stage 2 (*P* = 0.010), and it was higher in stage 2 than that in stage 1 (*P* = 0.029). However, the levels of depression, anxiety, and fear of COVID-19 were significantly lower in stage 3c relative to stage 3a (*Ps* < 0.05). It indicates that psychological distress varies among different stages.

### Univariate linear regression analysis results

Table 1 displays the results of univariate linear regression. Female, having a mediocre financial situation and poor physical health, experiencing financial losses, and having residual symptoms after the acute syndrome (*n* = 802) were all linked to higher levels of depression, anxiety, and fear of COVID-19 in all participants. Age was significantly associated with greater levels of fear of COVID-19. Booster vaccination was significantly associated with fear of COVID-19. Infection of relatives and friends was significantly associated with depressive and anxiety symptoms. Nevertheless, there was no significant association between religiosity or medication preparation and any form of psychological distress.

### Residual symptoms have major effect on psychological distress

Multiple linear regression analysis was conducted to determine the contribution of each variable to depression, anxiety, and fear of COVID-19 (Table 2). Older age was significantly associated with lower levels of depressive and anxiety symptoms. Moreover, gender, financial situation, and financial losses after lifting the COVID-19 policy were related to the fear of COVID-19. In addition, financial losses during the COVID-19 pandemic were associated with depressive symptoms. Furthermore, physical health and residual symptoms were significantly associated with depression, anxiety, and fear of COVID-19 after relief from an acute COVID-19 syndrome. The standardized coefficient β of residual symptoms for psychological distress was the highest among all variables, indicating that it has major contributions to depression, anxiety and fear of COVID-19.

**Table 2:** Regression models explore residual symptoms’ contribution in explaining psychological distress, above and beyond other demographic characteristics, and COVID-19-related information (*n* = 802)

Variables	Depressive symptoms^a^		Anxiety symptoms^a^		Fear of COVID-19^a^	
	β	R^2^	β	R^2^	β	R^2^
Age	-0.09*	0.12	-0.12**	0.09	0.09	0.12
Gender	0.01		0.01		0.10**	
Financial situation	-0.07		-0.06		-0.10**	
Physical health	0.13***		0.11**		0.09**	
COVID-19 Vaccination	-0.05		-0.04		-0.01	
Financial losses during the COVID-19 pandemic	0.11*		0.06		-0.02	
Financial losses after lifting the COVID-19 policy	0.05		0.07		0.16**	
Infections in relatives and friends	0.03		0.01		-0.03	
Residual symptoms	0.23***		0.21***		0.14***	

a: Multiple linear regression analysis, β: Standardized coefficient, R^2^: Coefficient of Determination, COVID-19: The coronavirus disease 2019, *: *P* < 0.05, **: *P* < 0.01, ***: *P* < 0.001

### Residual symptoms differentially affect risk perception and psychological distress at acute phase and chronic phase of COVID-19

**Fig. 2:**
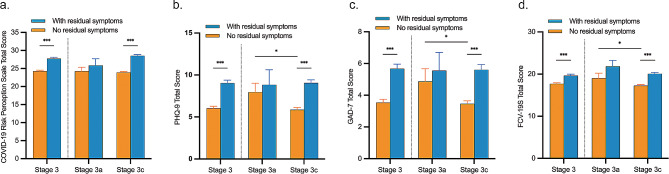
Residual symptoms differentially affect risk perception and psychological distress at acute phase and chronic phase after COVID-19 acute remission. Risk perception (**a**), depressive symptoms (**b**), anxiety symptoms (**c**) and fear of COVID-19 (**d**) were assessed by scales of COVID-19 risk perception, PHQ-9, GAD-7 and FCV-19S, respectively. Stage 3a represents acute phase of stage 3, and stage 3c represents chronic phase of stage 3. Values represent mean ± SEM. *: *P* < 0.05, ***: *P* < 0.001 by Student’s t-test

Participants with residual symptoms had significantly higher (*Ps* < 0.001) levels of risk perception and psychological distress compared to those without residual symptoms after COVID-19 infection (Fig. 2a, b, c and d). No significant difference in risk perception and psychological distress was detected between the subgroups with and without residual symptoms at stage 3a, although the trends were observed. Importantly, levels of risk perception (28.54 ± 6.26 vs. 23.90 ± 6.71), depression (9.05 ± 6.05 vs. 5.87 ± 5.39), anxiety (5.61 ± 5.26 vs. 3.46 ± 4.22), and fear of COVID-19 (20.05 ± 5.93 vs. 17.26 ± 5.88) were all significantly higher (*Ps* < 0.001) in the subgroup with residual symptoms than those in the subgroup without residual symptoms at stage 3c. Furthermore, among the participants without residual symptoms, lower levels of psychological distress were found at stage 3c compared with those at stage 3a (*Ps* < 0.05).

### The mediating role of risk perception in the relationship between residual symptoms and psychological distress

We conducted partial correlation analyses to examine the relationship between interested variables (Supplementary Table S1). Results showed that there were significant correlations between residual symptoms, risk perception of COVID-19, psychological distress, and IU after adjusting for age, gender, financial situation, and physical health (*Ps* < 0.01).

To test a simple mediating model, Model 4 in SPSS macro PROCESS was applied [45]. Residual symptoms had significant effects on depressive symptoms (β = 0.512, *P* < 0.001), anxiety symptoms (β = 0.458, *P* < 0.001), and fear of COVID-19 (β = 0.318, *P* < 0.001) when age, gender, financial situation, and physical health were controlled (Supplementary Table S2). When the risk perception was included, the direct effects of residual symptoms on depressive symptoms (β = 0.342, *P* < 0.001) and anxiety symptoms (β = 0.300, *P* < 0.001) remained significant, while the effect on fear of COVID-19 was no longer significant (β = 0.047, *P* = 0.475). Residual symptoms significantly affected risk perception (β = 0.497, *P* < 0.001). Moreover, risk perception was significantly associated with depressive symptoms (β = 0.341, *P* < 0.001), anxiety symptoms (β = 0.316, *P* < 0.001), and fear of COVID-19 (β = 0.546, *P* < 0.001). Additionally, the indirect effects of residual symptoms on depressive symptoms (estimate = 0.169, 95% CI = [0.110, 0.232]), anxiety symptoms (estimate = 0.157, 95% CI = [0.101, 0.221]), and fear of COVID-19 (estimate = 0.271, 95% CI = [0.188, 0.359]) were all significant, accounting for 33.01%, 34.28%, and 85.22% of each total effect, respectively.

### Intolerance of uncertainty moderates the effect of risk perception on psychological distress

**Fig. 3:**
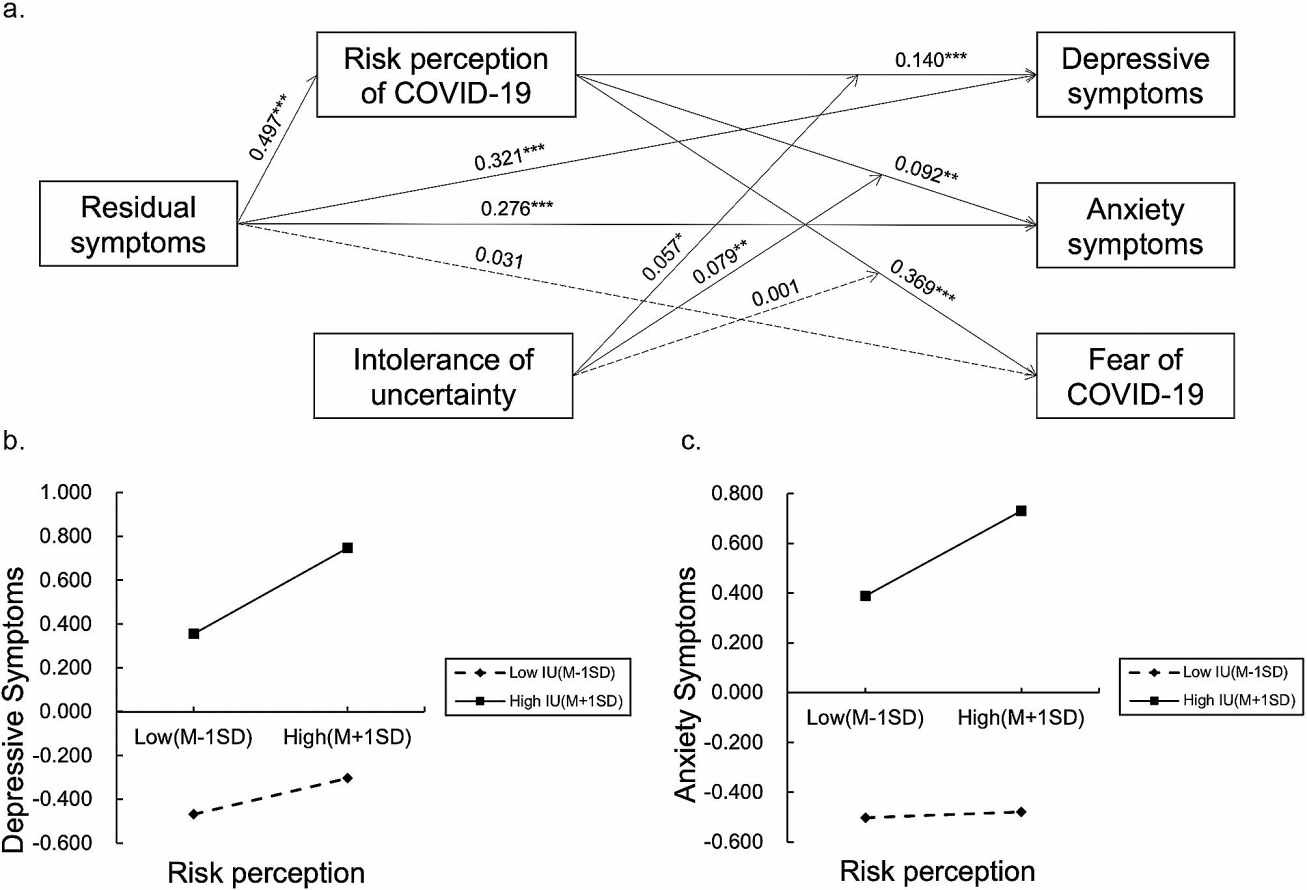
The moderated-mediation effects of risk perception and intolerance of uncertainty between residual symptoms and psychological distress. (**a**) Statistical diagram of the moderated mediation model. The numbers on the line are standardized regression coefficients. Non-significant paths are shown as dashed lines. (**b**) Intolerance of uncertainty (IU) moderated the relation between risk perception and depressive symptoms by simple slope analysis. (**c**) Intolerance of uncertainty (IU) moderated the relation between risk perception and anxiety symptoms by simple slope analysis. M-1SD represents Mean-1 SD. M + 1SD represents Mean+1 SD. *: *P* < 0.05, **: *P* < 0.01, ***: *P* < 0.001

To further investigate the moderating effect of IU in the mediation model, Model 14 of the SPSS macro PROCESS was applied [45]. The results of moderated mediation analyses were presented in Tables 3and Fig. 3a. When IU was introduced in the model, it showed positive direct effects on depressive symptoms (β = 0.468, *P* < 0.001), anxiety symptoms (β = 0.524, *P* < 0.001), and fear of COVID-19 (β = 0.408, *P* < 0.001). Moreover, the interaction term of risk perception and IU had a significant effect on depressive symptoms (β = 0.057, *P* = 0.015) and anxiety symptoms (β = 0.079, *P* = 0.001), respectively, but not on fear of COVID-19 (β = 0.001, *P* = 0.952). These results suggest that IU played a moderating role in the associations of risk perception with depressive symptoms as well as anxiety symptoms, but not with fear of COVID-19. Additional simple slope tests revealed that higher levels of risk perception were associated with higher levels of depressive and anxiety symptoms among individuals with high IU (b_simple_ = 0.197 and 0.171, *Ps* < 0.001) (Fig. 3b and c). However, the effect of risk perception on depressive symptoms was weaker among the individuals with low IU (b_simple_ = 0.083, *P* = 0.038), while its effect on anxiety became non-significant (b_simple_= 0.012, *P* = 0.753).

**Table 3:** The moderating role of IU in the mediating effect of risk perception on the relation between residual symptoms and psychological distress (*n* = 802)

Model 14	Risk perception of COVID-19		Depressive symptoms		Anxiety symptoms		Fear of COVID-19	
Variables	β	t	β	t	β	t	β	t
Constant	-0.596	-2.501*	0.118	0.560	0.191	0.916	-0.233	-1.216
Age	0.008	2.835**	-0.007	-2.932**	-0.008	-3.401**	0.006	2.890**
Gender	0.180	2.477*	-0.012	-0.185	0.001	0.018	0.143	2.433*
Financial situation	-0.428	-4.050***	-0.091	-0.962	-0.061	-0.649	-0.083	-0.966
Physical health	0.412	3.294**	0.163	1.453	0.041	0.368	-0.049	-0.483
Residual symptoms	0.497	6.993***	0.321	4.977***	0.276	4.317***	0.031	0.526
Risk perception of COVID-19			0.140	4.076***	0.092	2.700**	0.369	11.797***
IU			0.468	14.422***	0.524	16.331***	0.408	13.824***
Risk perception of COVID-19 x IU			0.057	2.446*	0.079	3.422**	0.001	0.060
R^2^	0.153		0.370		0.387		0.472	
F	28.724		58.151		62.564		88.673	

β: Standardized coefficient, R^2^: Coefficient of Determination, COVID-19: The coronavirus disease 2019, IU: Intolerance of uncertainty, *: *P* < 0.05, **: *P* < 0.01, ***: *P* < 0.001

## Discussion

The discrepancy of psychological distress levels at different stages of COVID-19 infection was revealed in this study. Moreover, residual symptoms were significantly associated with psychological distress (i.e., depression, anxiety, fear of COVID-19) among the sample of Chinese population following COVID-19 infection, which played the most important role in exacerbating and prolonging psychological distress. Furthermore, we found that risk perception of COVID-19 played a mediating role between residual symptoms and psychological distress, while IU moderated this mediating effect.

The first critical finding was that psychological distress varied at different stages from the high-risk period to the first month after infection. The study is unique in distinguishing different infection stages. The results suggest that COVID-19 symptoms may affect mood, even if it is not fully diagnosed. This finding is consistent with previous work [46, 47]. During the period of COVID-19 infection, the heightened psychological distress is a justifiable response to the risk [28, 48] and uncertainty [26, 49] surrounding the virus. However, the subgroup analysis indicated that psychological distress was significantly lower at stage 3c than that at stage 3a. The results suggest that the impact of COVID-19 infection on mild mental health outcomes is transient and self-recovered. It is consistent with previous studies that psychological distress tends to be decreased over time following the acute phase of infection, eventually returning to the baseline level [50–52].

Secondly, we found that a considerable proportion of patients have residual symptoms, which were significantly associated with higher levels and longer duration of psychological distress. Our data are in line with previous studies. For example, Matsumoto et al. found that about half of people had residual physical symptoms after COVID-19 and that these symptoms may lead to the onset of mental disorders [19]. Malik et al. discovered that post-COVID syndrome is a significant risk factor for mental health sequelae [21]. In addition, patients who have been hospitalized with COVID-19 may experience long-lasting mental health effects even after being cleared of the virus. These effects can be attributed to the persistence of physical symptoms and the duration of their illness [51, 53]. The study also revealed that residual symptoms in infected individuals led to the development of a persistent risk perception that was hard to be alleviated. It could be attributed to the fact that these symptoms reinforced the individual’s worry and distress regarding potential reinfection, relapse, or job performance [54]. Prolonged residual symptoms [23] indicate the possibility of long-term mental health impairment. Therefore, policymakers and clinicians should prioritize managing residual symptoms after acute COVID-19 syndrome from the outset and address any associated psychological distress.

Thirdly, we explored the mediating role of risk perception in the association between residual symptoms and psychological distress. We found that residual symptoms were associated with elevated risk perception. These symptoms might make one feel that the infection becomes more severe and uncontrollable, which leads to increased insecurity and uncertainty about the future [25]. Our study highly supports that an individual’s risk perception of COVID-19 is associated with experiencing symptoms of the disease [55], exposure to media coverage about COVID-19 [56, 57], and depression [48] and anxiety [58, 59], ultimately impacting an individual’s mental well-being during a public health crisis [28]. Moreover, these senses of insecurity and uncertainty may further result in an increased perception of risk. Since the way media reports on COVID-19 may affect people’s risk perception, misleading information can elevate perceived risk and contribute to psychological distress [56, 59]. This impact may be especially significant for those with problematic social media use as they spend quite much time on social media and are vulnerable to the risk of mental health issues [60]. In contrast, positive information was associated with less perceived risk and lower anxiety [58]. Therefore, media reports should be clear, rational, and positive, which may help people better understand the process of COVID-19 and reduce excessive risk perception.

We further examined the moderating effect of IU on the associations of risk perception with depression and anxiety. Among the individuals with high IU, those with higher levels of risk perception were more likely to experience depression and anxiety. Conversely, this association was less evident among those with low IU. Andrews et al. also discovered that IU had a significant effect on the regulation of psychological distress amidst the COVID-19 pandemic [61]. IU was recognized as a dispositional incapacity [31]. Individuals with high IU may have difficulty in coping with insecurity and uncertainty during COVID-19 infection. Therefore, high IU may amplify the effect of risk perception on depressive and anxiety symptoms, contributing to psychological distress. In contrast, the effect of risk perception on depression and anxiety tended to be non-significant in the participants with low IU, which indicated that IU plays a key role in the emergence of psychological distress in COVID-19 survivors. Previous studies have also found that IU was directly associated with higher depression and anxiety [25, 26], which is consistent with our study. Therefore, intervention in risk perception and IU may alleviate psychological distress. For instance, cognitive-behavioral therapy (CBT) might be helpful to affect one’s perceived risk and uncertainty [62].

Despite the moderated effects found in models of depression and anxiety, a distinct pattern emerged in the associations with fear of COVID-19. The mediating effect of risk perception appeared to play a major role in the association between residual symptoms and fear of COVID-19, while the moderating effect of IU was not evident. The positive association between risk perception and fear of COVID-19 aligns with previous findings [63, 64]. High risk perception and compromised health status have been reported to be robust contributors to heightened fear of COVID-19 [65]. A decline in physical health status may alter the perception of the COVID-19 risk, consequently contributing to higher levels of fear of the disease. Fear is typically an emotion directing towards a specific object, serving to motivate people to avoid potential danger. In contrast to the effects in depression and anxiety models, IU did not modulate the relationship between risk perception and fear of COVID-19. This disparity suggests that the fear of COVID-19 may directly stem from specific and definite negative consequences of COVID-19, such as residual symptoms and distressing experiences.

Compared with manifestations of COVID-19 in its presymptomatic and prodromal periods, the emergence of post-COVID syndrome has become a more prevalent public health concern in the current period. As some residual symptoms may be persisted and evolve into post-COVID syndrome, there is a critical transition phase between the acute phase and post-COVID syndrome. Individuals in this transition phase may be especially at risk of psychological distress, as they experience more severe physical symptoms than post-COVID syndrome and worry about the risk of transforming to post-COVID syndrome. Since the psychological distress has been identified as a risk factor of post-COVID syndrome [66, 67], our research sheds light on the potential mediator and moderator in the relationship between residual symptoms and psychological distress. As discussed above, these psychological factors (i.e., risk perception and IU) have been identified as potential targets for psychological intervention, offering the possibility to mitigate psychological distress and the risk of post-COVID syndrome. Therefore, this study holds significant implications for coping with this critical transition phase, thereby addressing a notable gap in the existing body of COVID-19 research.

To the best of our knowledge, this study is the first to investigate the possible psychological differences from never infected with COVID-19 stage to the chronic phase of COVID-19, and to explore the effects of residual symptoms on psychological distress and underlying mechanism. Our study identifies the significant impact of residual symptoms on psychological distress and the key role of risk perception and uncertainty intolerance (IU). It indicated that two psychological structures can be intervened in. However, the present study has several limitations. First, the generalizability of the study’s findings may be constrained by the use of convenience sampling and the limited number of elderly participants. As such, caution is warranted when extending the conclusions beyond the sampled population. Second, the limited number of participants within a week after COVID-19 infection should be considered when interpreting the subgroup analysis results. Third, this study follows a cross-sectional design, which necessitates longitudinal cohort studies to confirm the causality and long-term dynamics of residual symptoms.

## Conclusion

A considerable proportion of patients experience residual symptoms after the acute phase of COVID-19, which have a significant impact on psychological distress. Risk perception and intolerance of uncertainty play a moderated-mediation role in the association between residual symptoms and depression/anxiety. It highly suggests that effective treatment for residual symptoms, maintaining appropriate risk perception and improving intolerance of uncertainty are critical strategies to alleviate COVID-19 infection-associated psychological distress.

### Electronic supplementary material

Below is the link to the electronic supplementary material.

Supplementary Material 1

## Data Availability

All data used and/or analyzed in the present study are available from the corresponding authors upon request (Email: wuyili@wmu.edu.cn). They are not publicly available, in accordance with the Ethics Review Authority.

## Abbreviations

COVID-19: The coronavirus disease 2019
PHQ-9: Patient Health Questionnaire-9
GAD-7: Generalized Anxiety Disorder-7
FCV-19S: Fear of COVID-19 Scale
IU: Intolerance of uncertainty
IUS-12: Intolerance of Uncertainty Scale-12
ANCOVAs: Analyses of covariance
CI: Confidence interval
SD: Standard deviation
