# Supplementary material for: The moderated-mediation role of risk perception and intolerance of uncertainty in the association between residual symptoms and psychological distress: a cross-sectional study after COVID-19 policy lifted in China
Source: BMC Psychiatry. 2024 Feb 16;24:136. doi: 10.1186/s12888-024-05591-9 (PMC10874086; doi:10.1186/s12888-024-05591-9)

Supplementary Table S1

Spearman correlations between residual symptoms, risk perception of COVID-19, psychological distress, and IU adjusting for age, gender, financial situation, and physical health.

|  | Variables | 1 | 2 | 3 | 4 | 5 | 6 |
| --- | --- | --- | --- | --- | --- | --- | --- |
| 1 | Residual symptoms | 1 |  |  |  |  |  |
| 2 | Risk perception of COVID-19 | 0.260*** | 1 |  |  |  |  |
| 3 | Depressive symptoms | 0.245*** | 0.336*** | 1 |  |  |  |
| 4 | Anxiety symptoms | 0.207*** | 0.288*** | 0.785*** | 1 |  |  |
| 5 | Fear of COVID-19 | 0.143*** | 0.505*** | 0.361*** | 0.439*** | 1 |  |
| 6 | IU | 0.112** | 0.365*** | 0.490*** | 0.524*** | 0.537*** | 1 |

IU: Intolerance of uncertainty, ** *P* < 0.01, *** *P* < 0.001.

Supplementary Table S2

The mediating effect of risk perception of COVID-19 on the relation between residual symptoms and psychological distress (n = 802).

| Model 4 | Risk perception of COVID-19 | | Depressive symptoms | | Anxiety symptoms | | Fear of COVID-19 | |
| --- | --- | --- | --- | --- | --- | --- | --- | --- |
| Variables | β | t | β | t | β | t | β | t |
| Constant | -0.596 | -2.501* | -0.009 | -0.037 | 0.055 | 0.224 | -0.366 | -1.713 |
| Age | 0.008 | 2.835** | -0.010 | -3.835*** | -0.012 | -4.300*** | 0.004 | 1.520 |
| Gender | 0.180 | 2.477* | -0.048 | -0.665 | -0.043 | -0.570 | 0.119 | 1.831 |
| Financial situation | -0.428 | -4.050*** | -0.086 | -0.806 | -0.053 | -0.483 | -0.087 | -0.911 |
| Physical health | 0.412 | 3.294** | 0.419 | 3.332** | 0.336 | 2.608 | 0.147 | 1.303 |
| Residual symptoms | 0.497 | 6.993*** | 0.342 | 4.676*** | 0.300 | 4.004*** | 0.047 | 0.714 |
| Risk perception of COVID-19 |  |  | 0.341 | 9.614*** | 0.316 | 8.707*** | 0.546 | 17.218*** |
| R^2^ | 0.153 | | 0.186 | | 0.152 | | 0.341 | |
| F | 28.724 | | 30.296 | | 23.752 | | 68.710 | |

β: Standardized coefficient, R^2^: Coefficient of Determination, COVID-19: The coronavirus disease 2019, *: P < 0.05, **: P < 0.01, ***: P < 0.001.

Supplementary Figure S1: Proposed stages of COVID-19 infection. Stage 3a and stage 3c represent the acute phase and chronic phase of stage 3, respectively.


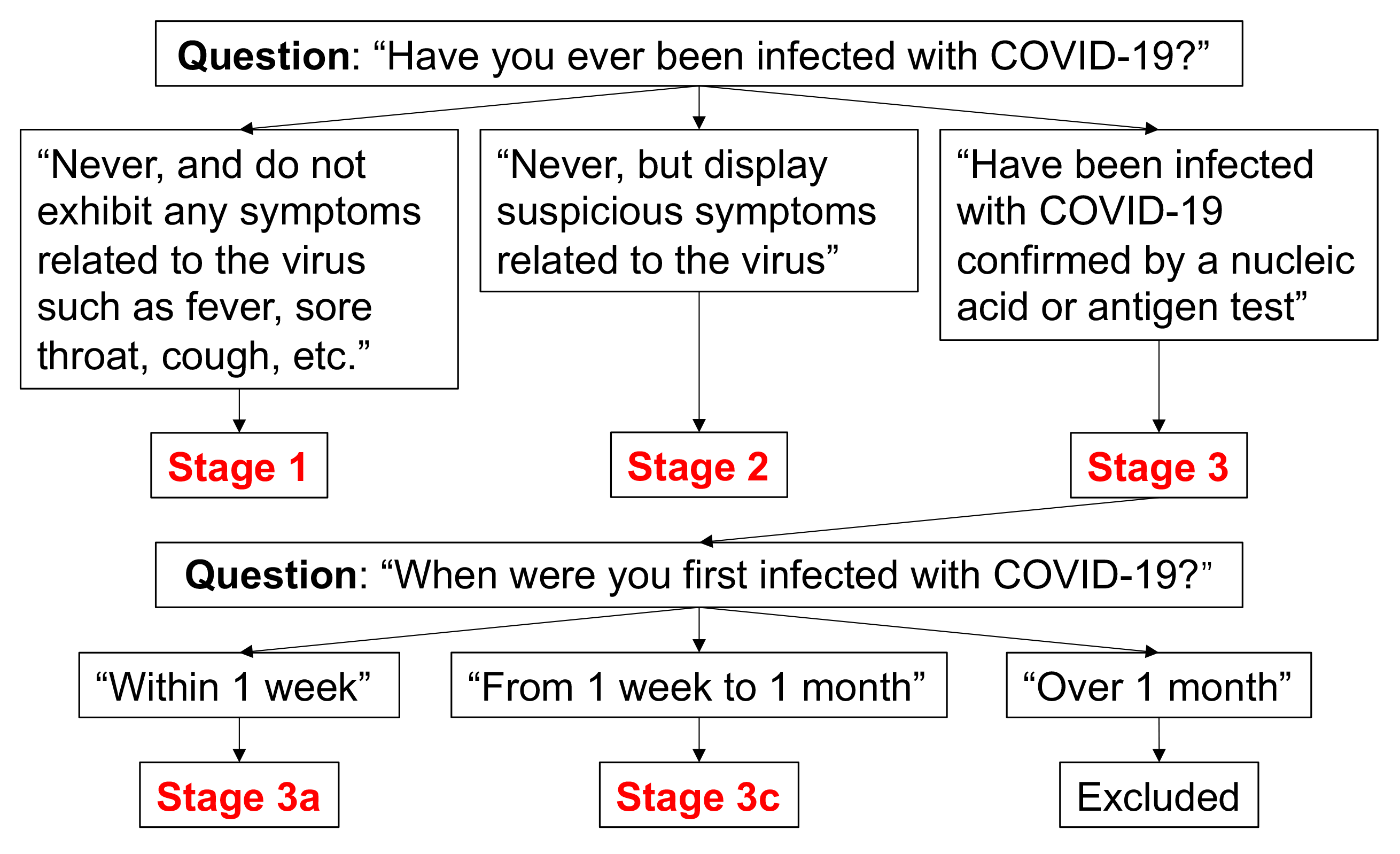

Supplement: Supplementary file 1 — Supplementary Material 1 [file 12888_2024_5591_MOESM1_ESM.docx]
